# Palliative care knowledge and attitudes towards end-of-life care of nurses in tertiary hospitals

**DOI:** 10.4102/curationis.v48i1.2734

**Published:** 2025-07-28

**Authors:** Eilean R. Lazarus, Joshua K. Muliira, Omar Al-Zaabi, Murtadha K. Al-Khabori, Mudhar M. Al Adawi, Qasim Al Mamari

**Affiliations:** 1 Department of Adult Health and Critical Care, College of Nursing, Sultan Qaboos University, Muscat, Oman; 2 Department of Hematology, Sultan Qaboos University Hospital, Muscat, Oman; 3 Royal Hospital, Muscat, Oman; 4 Medical Ward, Sultan Qaboos University Hospital, Muscat, Oman

**Keywords:** attitude, end-of-life care, knowledge, nursing, Oman, palliative care

## Abstract

**Background:**

Efforts to increase access to palliative care (PC) must consider healthcare providers’ level of knowledge and attitude because both affect the quality of services. Nurses play essential roles in the delivery of PC, including end-of-life care.

**Objectives:**

The study aimed to describe nurses’ readiness to provide PC in Oman by measuring PC knowledge and attitudes towards end-of-life care.

**Method:**

A cross-sectional design was used. The Palliative Care Knowledge Questionnaire and the Modified Frommelt Attitude towards the Care of the Dying Scale were used to collect data from nurses (*n* = 1469) practising in government tertiary hospitals across Oman.

**Results:**

The nurses exhibited suboptimal PC knowledge and positive attitudes towards end-of-life care. Significant differences were noted in some domains of knowledge and attitudes of nurses with different levels of professional experience. Attitude towards end-of-life care was associated with experience, caring for a dying family member, education and training in PC, religiosity, spirituality and age, but none was a significant predictor. The predictors of PC knowledge were gender, caring for a dying family member and education and training in PC.

**Conclusion:**

Nurses in tertiary care settings in Oman have positive attitudes towards end-of-life care, but they lack optimal PC knowledge. The gaps in knowledge could be attributed to professional education, training and experience.

**Contribution:**

The identified gaps in nurses’ PC knowledge could influence the provision of PC. Interventions are needed to sustain optimal knowledge and competencies to deliver quality PC to patients and their families.

## Introduction

Palliative care (PC) is a critical section of the healthcare system that helps enhance the quality of life of patients diagnosed with progressive illnesses and terminal illnesses and during end-of-life care (Zeru et al. 2020). The World Health Organization (2025) defines PC as an approach that improves the quality of life of patients and families facing problems associated with progressive or life-limiting illnesses. Palliative care prevents and relieves suffering through the early identification, correct assessment and treatment of pain and other problems, whether physical, psychosocial or spiritual (World Health Organization 2025). The PC care continuum includes three phases: PC, where the person is introduced to PC and his life expectancy is months to years; end-of-life care, when the person’s life expectancy is weeks to months and terminal care, provided during the patient’s last hour to days. Palliative care encompasses both end-of-life and terminal care. End-of-life care includes the provision of physical, emotional, social and spiritual support for patients and their families with the goals of controlling pain and other symptoms so the patient can be as comfortable as possible (Huffman & Harmer 2025).

However, it is important to note that access to PC services is inadequate in some countries. Globally, 29 million patients with life-limiting illnesses died and required PC in the year 2020, and the demand for PC is predicted to continue increasing because of the increasing prevalence of chronic diseases associated with ageing, cardiovascular disorders, renal failure, cancer and others (Getie et al. 2021). Recent studies show that many people requiring End-of-Life Care (EOLC) live in low- and middle-income countries (Getie et al. 2021; Mehrolhassani et al. 2020). For instance, the population requiring PC is increasing steadily in countries like India, Iran, Ethiopia, Sudan, Nigeria and the Gulf Cooperation Council countries such as Oman (Getie et al. 2021).

The other factors fuelling the demand for PC services include advancements in medical technology that have increased survival rates and the lifespan of terminally ill patients (Seven & Sert 2020). Such patients require continuous PC to address their needs related to pain and other distressing symptoms until the end of life (Seven & Sert 2020). Additionally, patients and their families encounter many challenges during progressive life-limiting illnesses because of the problems associated with pain, fear of impending death, disabilities and physical and emotional distress (Muliira, Kizza & Al-Kindi 2024; Seven & Sert 2020). Therefore, PC is now recognised as an essential part of the healthcare provided in any country (Getie et al. 2021).

Palliative care is recognised as a type of specialised care focused on pain, other symptoms and the quality of life of patients with incurable conditions, terminal illnesses and end-of-life situations (Kim, Kim & Gelegjamts 2020). Nurses play a central role in delivering PC to patients with progressive life-limiting illnesses through assessment, medication administration, counselling, follow-up care, patient and family education and other interventions (Zeru et al. 2020). Nurses are usually the first healthcare personnel to recognise or receive information about the patient’s pain, suffering, symptoms and end-of-life situations (Mehrolhassani et al. 2020). The nurses provide the direct care needed to alleviate suffering, and this kind of care requires nurses working in tertiary care settings where most deaths take place to have adequate knowledge about PC and a positive attitude towards death (Seven & Sert 2020; Zeinah, Al-Kindi & Hassan 2013). Nurses with inadequate knowledge and negative attitudes towards PC may hinder quality PC for patients with chronic illnesses and during end-of-life situations (Getie et al. 2021). Thus, nurses must have adequate knowledge, understanding and favourable attitudes towards PC (Mehrolhassani et al. 2020; Zeru et al. 2020).

The current study was conducted in Oman, a country located in the Middle East and part of the World Health Organization’s Eastern Mediterranean Region (EMRO). Access to PC services is a major challenge in the EMRO since only 5% of the population has access (Pourghazian et al. 2022). Many countries in the EMRO have no national operational PC plans, or the plans have only been recently developed (Fadhil & Ghali 2021). It has been documented elsewhere that to enhance PC services at the national level, efforts need to consider the providers’ level of PC knowledge and attitude towards death because they both affect the quality of care (Etafa et al. 2020; Hao et al. 2021; Kim et al. 2020). Limited knowledge among providers, such as nurses, contributes to a lack of confidence in delivering PC services and increased feelings of stress and guilt while providing EOLC to patients and families (Altarawneh et al. 2023).

In addition to the lack of adequate access to PC in Oman, the prevalence of non-communicable diseases such as cancer is increasing, and such diseases are now a major cause of death (Al-Azri et al. 2021). On the other hand, available studies show that the knowledge and attitude towards PC among nursing and medical students in Oman are low (Al-Azri et al. 2021; Muliira, Lazarus & Mirafuentes 2023). No studies have focused on the PC knowledge and attitudes towards EOLC of nurses currently in clinical practice and responsible for caring for the increasing population with terminal illnesses in Oman. The current study aimed to describe nurses’ readiness to provide PC in Oman by measuring PC knowledge and attitudes towards end-of-life care. Understanding the PC knowledge and attitudes towards EOLC of nurses in Oman can help to establish gaps in knowledge and guide the development of interventions needed to enhance access to quality PC (Abu Sharour et al. 2021; Cegelka, Khan & Assaly 2019).

## Research design and methods

### Study design

The study used a cross-sectional design. A cross-sectional design is characterised by the collection of relevant data at a given point in time, and it is the most relevant design when describing knowledge and attitudes among health personnel (Kesmodel 2018).

### Setting

The study was conducted in government tertiary hospitals located in all the nine regions of Oman that have this level of hospital. The tertiary hospitals are the referral centres where patients with complex health problems from primary health centres and polyclinic are referred for specialised management. In Oman, all citizens receive free healthcare in all government healthcare facilities, including tertiary hospitals. The regions and hospitals from which the participants were recruited are summarised in Table 1.

**TABLE 1:** The regions and hospitals in Oman where the participants were recruited.

Region in Oman	Name of tertiary hospital	No. of nurses in the hospital	No. of participants eligible for the study	No. of participants returning completed questionnaire	
				*n*	%
Muscat	Khoula Hospital	1227	306	207	14.2
	Royal Hospital	1924	481	246	16.8
	Sultan Qaboos University Hospital	1129	644	206	14.0
Dhofar	H. As Sultan Qaboos Hospital	1031	257	109	7.4
Al Burayimi	Al Burayimi Hospital	263	65	61	4.2
Ad Dakhliyah	Nizwa Hospital	652	163	122	8.3
Al Batinah North	Sohar Hospital	830	207	181	12.3
Al Batinah South	Ar Rustaq Hospital	550	137	80	5.5
Ash Sharqiyah South	Sur Hospital	327	80	74	5.0
Ash Sharqiyah North	Ibra Hospital	401	101	94	6.4
Adh Dhahirah	Ibri Hospital	413	104	89	6.1

**Total**	–	**8747**	**2545**	**1469**	**57.7**

### Study population, sample and sampling method

The study focused on registered nurses working in tertiary hospitals in Oman. This population was targeted because, in Oman, patients with chronic illness are referred to tertiary hospitals for further symptom and disease management and experience their end of life in tertiary hospitals. Thus, nurses working on units that care for such patients are expected to be familiar with the principles of PC and to have favourable attitudes towards EOLC. Thus, the respondents were registered nurses employed in government tertiary care hospitals in Oman.

The required minimum sample for the study was 50% (*n* = 1273) of the eligible participants (*N* = 2545). The participants in each setting were selected using a consecutive sampling strategy (Thewes et al. 2018). Consecutive sampling is a non-probability sampling strategy where samples are chosen consecutively until the required number for the study is obtained (Thewes et al. 2018). Consecutive sampling was used because of its advantage of improving the sample’s representativeness of the target population.

The list of nurses in each unit was used to identify every nurse meeting the criteria of inclusion, and these were selected and approached to participate in the study. The inclusion criteria were having at least 1 year of clinical experience as a registered nurse, the ability to read and write in English and working on units that receive patients who may require PC, such as the emergency room, medical-surgical units, intensive care units and others. Nurses working in the operation theatres, psychiatric wards or units, outpatient or ambulatory services and day care centres not associated with chronic diseases and symptom management were excluded from the study. All eligible participants (*N* = 2545) were approached to participate in the study. A total of 1469 (*n* = 1469) returned the completed questionnaire and signed consent form (participation rate = 57.7%). The respondents were selected during the day, night and evening shifts. The number of nurses recruited from each hospital is presented in Table 1.

### Instruments used for data collection

The Palliative Care Knowledge Questionnaire (PEACE-Q) (Yamamoto et al. 2015) and the Modified Frommelt Attitude Toward the Care of the Dying Scale (FATCOD-B) (Barnett, Reed & Adam 2021; Chen et al. 2022; Kim et al. 2020) served as data collection instruments. The PEACE-Q and FATCOD-B have been used in Oman and showed good reliability (Muliira et al. 2023). The PEACE-Q scale has 34 items, and these evaluate healthcare providers’ understanding of aspects and concepts of PC such as the philosophy of PC, cancer pain management, side effects of opioid medications, nausea and vomiting, delirium, psychological distress, communication and higher scores indicating better PC knowledge (Yamamoto et al. 2015). Each item of the PEACE-Q requires a response of true or false, and this may be the correct or wrong answer depending on the item. The correct answer is given a score of one (1) and the wrong answer a score of zero (0). The total scores of the PEACE-Q range from 0 to 34. In the current study, the PEACE-Q had acceptable internal consistency with a Cronbach’s alpha of 0.77 and a KR-20 index of 0.79.

The FATCOD-B (30 items rated on a 5-point Likert scale) is a reliable scale used to measure attitudes towards caring for dying patients, with higher scores reflecting more positive attitudes (Barnett et al. 2021; Frommelt 1991; Nepal, Garbuja & Nepal 2021). The FATCOD-B has six sub-scales focusing on fear or malaise, the care of the family, communication, family as caring, relationships and active care, and high scores in each domain and the overall scale indicate a positive attitude. The overall FATCOD-B scores range from 30 to 150 (≥ 60 = favourable attitude and ≤ 60 = unfavourable attitude). The FATCOD-B scale demonstrated high reliability with a Cronbach’s alpha of 0.80.

## Data collection

After approval of the study, the researchers approached each hospital’s nursing director to explain the study’s aim and objectives and the data collection process. The research assistants (nurses excluded from participating in this study) were selected and permitted to approach colleagues in the respective hospitals to collect data during the day, night and evening shifts. The research assistants administered the study consent form and questionnaire to participants who met the inclusion criteria. The participants were identified from the lists (sampling frame) provided by the directorate of nursing of each hospital. The research assistants followed up with the participants using verbal reminders to collect the completed consent forms and questionnaires. Because of the patient care activities, the participants had 4 hours to complete the questionnaire. All administered questionnaires were collected on the same day, whether completed or left blank.

### Data management and analysis

The completed questionnaires were checked for missing data immediately at the point of data collection, and participants were asked to clarify the gaps. A codebook to enter data in the Statistical Packages for Social Sciences software program version 29 (SPSS) was developed to show data definitions, abbreviations and a range of possible numerical values for the different variables. The data were entered into an electronic SPSS file before cleaning and analysis. During data cleaning, the investigators looked for consistency and accuracy and checked the values of the variables. Frequency tables were run to monitor outliers. Where outliers existed, the investigators checked the original data to verify the presence of such values and confirm whether they were because of inherent variability within the data. Once data were verified, normality tests and Cronbach’s alpha were calculated to assess the internal consistency and reliability of the scales used in the study.

Descriptive statistics were used to analyse the data. The nurses were categorised into three groups according to their level of clinical experience (Level 1 = ≤ 5 years, Level 2 = 6–10 years and Level 3 = ≥ 10 years). The one-way Analysis of Variance (ANOVA) was used to determine the differences in the means of the variables. To analyse the pattern of differences between means, the ANOVA was followed by a specific pairwise comparison technique called the least significant difference (LSD) test. The LSD test can compute the smallest significant difference between three or more means, and it confirms any significant differences larger than the LSD. Additionally, the main variables (PC knowledge and attitude towards EOLC) were measured on continuous scales. Multiple linear regression analysis was used to determine the predictors of PEACEQ and FATCOD-B scores. The level of significance for all analyses was set at ≤ 0.05.

### Ethical considerations

The study was reviewed and approved by Sultan Qaboos University, the College of Nursing Research and Ethics Committee (SQU-EC/283/2022) and the Ministry of Health’s Research and Ethics Committee in Oman (MoH/CSR/22/26113). Before data collection, participants were informed about the objectives, procedures, potential benefits, risks and their rights during the study. The participants were required to read and sign the study consent form. A copy of the consent form was given to the participants who completed the study questionnaire. Participant confidentiality was ensured throughout the study by not collecting personal identifiers and using study serial numbers to label and track the questionnaires. Data were securely stored in password-locked computer files and were only accessed by the investigators involved in the study.

## Results

### Description of the sample

The nurses (*n* = 1469) who participated in the study had a mean age and clinical experience of 35.19 ± 6.70 years and 11 ± 6.50 years, respectively. The other participant characteristics are summarised in Table 2 according to the nurses’ level of clinical experience (Level 1 ≤ 5 years, Level 2 = 6–10 years and Level 3 ≥ 11 years). The lowest clinical experience was 1 year, and the highest clinical experience was 44 years. The participants worked in different units caring for paediatric and adult patients who required medical and surgical interventions for acute and chronic problems. Most participants across all experience levels were female (>82%), Omani (46.8%), and had a bachelor’s degree (54.5%). Many nurses had never been involved in caring for a family member with cancer (66.6%), did not receive education and training focusing on PC during nursing school (75%) and had not attended continuing education on PC after graduation (81%).

**TABLE 2:** Characteristics of the participants.

Characteristic	Level 1 (*n* = 298)			Level 2 (*n* = 400)			Level 3 (*n* = 771)			Total sample (*N* = 1469)			Test	
	*n*	%	Mean ± s.d.	*n*	%	Mean ± s.d.	*n*	%	Mean ± s.d.	*n*	%	Mean ± s.d.	*X*	*p*
Gender													3.34	0.101
Male	52	17.4	-	70	17.5	-	108	14.0	-	230	15.7	-	-	-
Female	246	82.6	-	330	82.5	-	663	86.0	-	1239	84.3	-	-	-
Nationality													256.07	0.001
Philippines	4	1.3	-	31	7.8	-	104	13.3	-	139	9.5	-	-	-
Oman	48	16.1	-	199	49.8	-	441	57.2	-	688	46.8	-	-	-
India	246	82.6	-	170	42.4	-	226	56.5	-	642	43.7	-	-	-
Marital status													104.50	0.001
Married	186	62.5	-	329	82.3	-	676	87.7	-	1191	81.1	-	-	-
Single	111	37.2	-	65	16.2	-	86	11.2	-	262	17.8	-	-	-
Divorced	1	0.3	-	6	1.5	-	9	1.1	-	16	1.1	-	-	-
Professional level of education													242.53	0.001
Associate	40	13.4	-	166	41.5	-	421	54.6	-	627	42.7	-	-	-
Bachelors	250	83.9	-	228	57.0	-	323	41.9	-	801	54.5	-	-	-
Masters	8	2.7	-	6	1.5	-	27	3.5	-	41	2.8	-	-	-
Age (years)†	-	-	27.64 ± 2.78	-	-	32.78 ± 3.50	-	-	39.36 ± 5.82	-	-	35.19 ± 6.70	-	0.001
Has been involved in providing care for a person with cancer in their family													12.04	0.002
Yes	90	30.2	-	112	28.0	-	288	37.4	-	490	33.4	-	-	-
No	208	69.8	-	288	72.0	-	483	62.6	-	979	66.6	-	-	-
Has been involved in caring for dying close family member													9.11	0.011
Yes	113	37.9	-	189	47.3	-	369	47.8	-	671	45.7	-	-	-
No	185	62.1	-	211	52.8	-	402	52.2	-	798	53.3	-	-	-
Received education and training on palliative care while studying in nursing school													13.84	0.001
Yes	94	31.5	-	110	27.5	-	164	21.2	-	368	25.1	-	-	-
No	204	68.5	-	290	72.5	-	607	78.8	-	1101	74.9	-	-	-
Received education and training on palliative care after graduation as part of a continuing education programme													2.19	0.701
Yes	49	16.4	-	80	20.0	-	149	19.3	-	278	18.9	-	-	-
No	249	83.6	-	320	80.0	-	622	80.7	-	1191	81.1	-	-	-

Note: Level 1 Experience of ≤ 5 years, Level 2 Experience of 6–10 years, Level 3 Experience of ≥ 11 years.

s.d., standard deviation; LSD, least significant difference.

† , *F* = 721.87; one way analysis of variance; LSD test: Level 1 versus Level 2, p < 0.001; Level 1 versus Level 3, p < 0.001; Level 2 versus Level 3, *p* < 0.001.

### Palliative care knowledge

Table 3 summarises the nurses’ knowledge of PC. The mean score was 21.94 (s.d. = 3.59), equivalent to 64.5% correct responses. This level of PC knowledge is moderate. The ranking of the mean scores for the domains of PC knowledge shows that nurses were most knowledgeable about psychological distress, nausea and vomiting, communication, community PC and the philosophy of PC. There was no significant difference in the mean scores across groups (*p* = 0.271). The one-way ANOVA results showed statistically significant differences in the level of PC knowledge between groups of nurses according to their level of experience. The differences were in the mean scores for the domains of side effects of opioids (*F* = 3.05, *p* = 0.048), dyspnoea (*F* = 5.79, *p* = 0.003), psychological distress (*F* = 5.76, *p* = 0.003) and communication (*F* = 3.97, *p* = 0.019).

**TABLE 3:** Oman nurses’ palliative care knowledge (Palliative Care Knowledge Questionnaire scores).

Sub-scale	Rank	Mean ± s.d.				*F*	*p* *

Total	Level 1 (*n* = 298)	Level 2 (*n* = 400)	Level 3 (*n* = 771)
Philosophy of PC	4	2.65 ± 0.08	2.64 ± 0.91	2.65 ± 0.83	2.66 ± 0.82	0.75	0.928
Cancer pain	6	4.77 ± 1.59	4.85 ± 1.85	4.81 ± 1.56	4.72 ± 1.50	0.91	0.403
Side effects of opioids	5	1.87 ± 0.72	1.78 ± 0.81	1.89 ± 0.66	1.90 ± 0.71	3.05	0.048†
Dyspnoea	8	1.39 ± 0.84	1.26 ± 0.85	1.48 ± 0.83	1.40 ± 0.85	5.79	0.003‡
Nausea and vomiting	1	2.75 ± 0.83	2.40 ± 0.82	2.31 ± 0.92	2.30 ± 0.84	1.69	0.185
Psychological distress	1	2.75 ± 0.58	2.65 ± 0.68	2.78 ± 0.57	2.78 ± 0.55	5.76	0.003§
Delirium	7	1.50 ± 0.79	1.53 ± 0.68	1.44 ± 0.77	1.51 ± 0.77	1.50	0.224
Communication	2	2.44 ± 0.71	2.36 ± 0.78	2.41 ± 0.72	2.48 ± 0.67	3.97	0.019¶
Community PC	3	2.43 ± 0.72	2.17 ± 0.79	2.27 ± 0.69	2.27 ± 0.71	2.12	0.120
**Total PEACE-Q score**	**-**	**21.94 ± 3.59**	**21.64 ± 4.43**	**22.02 ± 3.51**	**22.01 ± 3.25**	**1.31**	**0.271**

Note: Level 1 Experience of ≤ 5 years, Level 2 Experience of 6–10 years, Level 3 Experience of ≥ 11 years.

s.d., standard deviation; PC, Palliative care; PEACE-Q, Palliative Care Knowledge Questionnaire; LSD, least significant difference.

* , One-way analysis of variance.

† , LSD test: Level 1 versus Level 2, p = 0.05; Level 1 versus Level 3, *p* = 0.016;

‡ , LSD test: Level 1 versus Level 2, *p* < 0.001; Level 1 versus Level 3, *p* = 0.016;

§ , LSD test: Level 1 versus Level 2, *p* = 0.005; Level 1 versus Level 3, *p* < 0.001;

¶ , LSD test: Level 1 versus Level 3, *p* = 0.008.

The LSD test revealed statistically significant differences in mean scores between Level 1 and Level 2 (*p* = 0.05) and between Level 1 and Level 3 (*p* = 0.016) on the knowledge about side effects of opioids. Additionally, significant differences were found in mean scores of dyspnoea between Level 1 and Level 2 (*p* < 0.001) and between Level 1 and Level 3 (*p* = 0.016). Regarding the mean scores for psychological distress, there were significant differences between Level 1 and Level 2 (*p* = 0.005) and between Level 1 and Level 3 (*p* < 0.001). Furthermore, significant differences were noted in mean scores of communications between Level 1 and Level 3 (*p* = 0.008). There were no significant differences in the mean scores on the sub-scales of the philosophy of PC, cancer pain, nausea and vomiting, delirium and community PC.

### Attitudes towards end-of-life care

The results of nurses’ attitudes towards EOLC measured using the FATCOD-B are presented in Table 4. The mean FATCOD-B score was 99.02 (s.d. = 11.33), suggesting favourable attitudes towards EOLC (score ≥ 60). The one-way ANOVA results show significant differences in the mean scores of the subdomains of the FATCOD-B across levels of clinical experience. The LSD test further revealed statistically significant differences in the mean scores for fear or malaise between Level 1 and Level 2 (*p* < 0.001) and between Level 1 and Level 3 (*p* < 0.001). Significant differences were also found in the mean scores for the subdomain of care of the family between Level 1 and Level 2 (*p* = 0.008), between Level 1 and Level 3 (*p* < 0.001) and between Level 2 and Level 3 (*p* = 0.023). There were also significant differences in the mean scores for the subdomain of communication between Level 2 and Level 3 (*p* < 0.001). Lastly, significant differences in the mean scores for the subdomain of the family as caring were found between Level 1 and Level 2 (*p* = 0.003), between Level 1 and Level 3 (*p* < 0.001) and between Level 2 and Level 3 (*p* = 0.05). There were no significant differences between groups in the overall FATCOB-B mean scores and the mean scores for the relationship and active care sub-scales.

**TABLE 4:** Oman nurses’ attitudes towards end-of-life care (Frommelt Attitude Toward the Care of the Dying Scale scores).

Sub-scale	Mean ± s.d.				*F*	*p* *
	Total	Level 1 (*n* = 298)	Level 2 (*n* = 400)	Level 3 (*n* = 771)		
Fear or malaise	27.39 ± 5.54	28.46 ± 5.15	26.94 ± 5.55	27.20 ± 5.63	7.43	< 0.001†
The care of the family	11.61 ± 2.55	11.01 ± 2.51	11.53 ± 2.64	11.88 ± 2.48	12.98	< 0.001‡
Communication	19.47 ± 3.52	19.39 ± 3.17	18.97 ± 3.58	19.76 ± 3.59	6.67	< 0.001§
Family as caring	11.75 ± 2.71	11.20 ± 2.77	11.71 ± 2.72	12.30 ± 2.63	13.06	< 0.001¶
Relationship	15.86 ± 2.52	16.04 ± 2.50	15.90 ± 2.49	15.78 ± 2.54	1.27	0.282
Active care	12.94 ± 2.70	13.04 ± 2.61	13.00 ± 2.74	12.86 ± 2.71	0.59	0.552
Total FATCOD-B score	99.02 ± 11.33	99.05 ± 10.94	98.03 ± 11.23	99.02 ± 11.33	2.32	0.099

Note: Level 1 Experience of ≤ 5 years, Level 2 Experience of 6–10 years, Level 3 Experience of ≥ 11 years.

s.d., standard deviation; FATCOD-B, Frommelt Attitude Toward the Care of the Dying Scale; LSD, least significant difference.

* , One-way analysis of variance.

† , LSD test: Level 1 versus Level 2, *p* < 0.001, Level 1 versus Level 3, *p* < 0.001;

‡ , LSD test: Level 1 versus Level 2, *p* = 0.008; Level 1 versus Level 3, *p* < 0.001; Level 2 versus Level 3, *p* 0.023;

§ , LSD test: Level 2 versus Level 3, *p* < 0.001;

¶ , LSD test: Level 1 versus Level 2, *p* = 0.003, Level 1 versus Level 3, *p* < 0.001; Level 2 versus Level 3, *p* 0.050.

### Factors associated with palliative care knowledge and attitude towards end-of-life care

The attitude towards EOLC was positively associated with years of clinical experience (*r* = 0.069, *p* = 0.008), caring for a dying family member (*r* = 0.071, *p* = 0.006) and receiving education and training in PC during nursing school (*r* = 0.053, *p* = 0.044). The participants’ age was the only non-modifiable factor associated with attitude towards EOLC. The attitudes towards EOLC were negatively associated with the level of self-rated religiosity (*r* = −0.62, *p* = 0.003) and spirituality (*r* = −0.052, *p* = 0.013). There were no significant predictors of FATCOD-B scores among the factors examined.

The non-modifiable factors associated with PC knowledge (PEACE-Q score) were age in years (*r* = 0.106, *p* < 0.001), gender (*r* = −0.063, *p* = 0.017) and marital status (*r* = −0.054, *p* = 0.040). Additionally, caring for dying close family members (*r* = 0.053, *p* = 0.043) and receiving education and training in PC during nursing school (*r* = 0.070, *p* = 0.007) were associated with better PC knowledge. Table 5 presents a regression analysis examining predictors of PC knowledge. The significant modifiable predictors of PC knowledge were the experience of caring for a dying family member (*p* = 0.012) and receiving education and training in PC during nursing school (*p* = 0.010). The three factors of gender, experience caring for a dying family member and receiving education and training in PC during nursing school explained 16% of the variance in PC knowledge (*R* = 0.128).

**TABLE 5:** Predictors of palliative care knowledge.

Factors	Unstandardised coefficients		Standardised coefficients beta	*t*	*p*	95% Confidence interval
	*B*	Standard error				
Constant (Enter methods)	23.43	1.049	-	22.34	< 0.001	21.38 to 25.49
Gender	−0.639	0.260	−0.065	−2.46	0.014	−1.15 to −0.129
Involved in caring for a dying family member	0.504	0.201	0.070	2.53	0.012	0.110 to 0.897
Received education and training programme in palliative care in nursing school	−0.612	0.237	−0.074	−2.59	0.010	0.00 to −0.148

## Discussion

The results about PC knowledge show that nurses working in Oman governmental tertiary care settings had moderate knowledge. These findings are similar to those of a study of Omani senior undergraduate nursing students (Muliira et al. 2023). The findings indicate a need for continuing education and training for nurses focusing on PC. Comparable results have been observed in studies conducted in Jordan, the USA and Australia (Al Qadire 2022). The findings of only moderate PC knowledge emphasise the imperative nature of ongoing professional development and continuing education as key strategies for enhancing the PC competencies of nurses (Al Qadire 2022; Cagle et al. 2020; Caruso et al. 2020; Ferrell et al. 2021).

Studies recently conducted in Canada and Mexico revealed similar results, as the present study, of no significant differences in PC knowledge scores across varying levels of clinical experience (Gaviria, Carreño-Moreno & Chaparro-Díaz 2023; Williams, Boumans & Luymes 2022). Studies conducted in Australia, South Korea, the USA and the United Kingdom also found knowledge gaps in aspects of PC, such as opioid management and symptom control, as shown in the present study (Bryk et al. 2023; Cagle et al. 2020; Caruso et al. 2020; Ferrell et al. 2021; Johnson et al. 2019). The findings also emphasise the challenge of ensuring that nurses possess the requisite knowledge and competencies to provide comprehensive end-of-life care to patients (Chan et al. 2020; Gerber et al. 2022; Uzelli Yilmaz et al. 2023). Regular efforts to ascertain and enhance PC knowledge and EOLC attitudes of nurses are needed to improve access to high-quality PC (Muliira et al. 2023).

The other findings show that nurses in Oman have favourable attitudes towards end-of-life care, and this is similar to reports from countries such as Jordan, Taiwan, Spain and Brazil (Al Qadire 2022; Chang et al. 2023; Hsieh 2020; Mengual et al. 2023; Teixeira et al. 2019). The findings highlight the commitment of nurses to delivering empathetic healthcare to patients (Hsieh 2020; Teixeira et al. 2019). It is important to acknowledge that attitudes towards end-of-life care can be influenced by cultural, religious and social values and norms (Hsieh 2020; Teixeira et al. 2019). These factors should also be considered from the patients and their families’ perspectives during EOLC. Additional studies of the determinants of positive attitudes will enable us to identify the factors that can be used to sustain positive attitudes and motivation for quality EOLC for patients.

Additionally, our results are like those of a study that described nurses’ knowledge and attitudes towards geriatric PC in Egypt (Fahim, Boughdady & Meawad 2023). The study in Egypt found that many nurses had favourable attitudes and moderate knowledge of PC (Fahim et al. 2023). Significantly, both studies revealed a possible deficiency in specialised education, as only a minority of nurses participated in education and training programmes that specifically addressed PC. Fahim et al. (2023) emphasise the significance of augmenting nurses’ education to enhance their competencies and attitudes towards end-of-life care. The study by Fahim et al. (2023) and the present study did not examine the curricula used for the education and training of nurses, and this is a gap that needs to be addressed by future studies.

The present study identified significant differences in attitudes across different levels of clinical experience, with more experienced nurses exhibiting more positive attitudes towards certain aspects of EOLC, such as fear or malaise, care of the family, communication and family as caring. In contrast, a study in Australia found a lack of association between clinical experience and attitudes towards EOLC (Caruso et al. 2020), suggesting that cultural or contextual differences may influence nurses’ attitudes. On the other hand, like in our study, in China, nurses with more experience tended to have more positive attitudes towards EOLC (Li, Kongsuwan & Yodchai 2023). Therefore, it is essential to consider differences in cultural factors and healthcare systems when examining nurses’ attitudes towards EOLC.

Studies conducted in Iran and Australia show that organisational support and the availability of resources play a crucial role in influencing the knowledge and abilities of nurses in PC (Nikkhah et al. 2022; Slater et al. 2021). The findings of the above studies emphasise the need to create supportive working environments that offer opportunities for ongoing professional education to enable nurses to deliver exceptional end-of-life care. Furthermore, a study conducted in the United Kingdom highlights the importance of interdisciplinary collaboration and adopting a comprehensive strategy for instruction and training to enhance PC delivery (Mason et al. 2018).

Nurses’ opinions about caring for patients at the end of life are significantly influenced by personal experiences, clinical experience and formal education. Prior studies have found a positive correlation between nurses’ perspectives about end-of-life care and exposure to the experience of caring for dying family members or personal contacts with major illnesses (Johnson et al. 2019; Rykkje et al. 2022). These results suggest that training in EOLC using *in situ* simulations and other active learning activities may enhance nurses’ PC and EOLC competencies.

### Limitations

The results of our study should be considered, given the limitations explained below. The use of the self-report method of data collection in an uncontrolled environment could underestimate or overestimate PC knowledge. Moreover, participants’ attitudes are bound to be influenced by the prevailing emotional state and recent experiences. The standardised study instruments used did not comprehensively measure factors such as access to continuing education, the PC content in the curriculum used to educate and train nurses, healthcare policies and PC protocols that could influence PC knowledge and attitudes towards EOLC. The sample had many nurses who were originally from India and the Philippines and who were educated in their respective countries. These nurses work in Oman, a majority Muslim country, and literature shows that internationally educated nurses may have attitudes and values about specific aspects of their work that are partly influenced by their native cultural values, beliefs and practices (Balante, Van den Broek & White 2021).

### Recommendations

Despite its limitations, the study findings lead us to recommend revision of the curriculum used for the education and training of nurses in Oman to ensure the inclusion of courses specifically addressing PC and EOLC. Moreover, nurses who are already in clinical practice can benefit from continuing education programmes that address PC and EOLC. We recommend the development of continuing education programmes for nurses currently in clinical practice to boost PC knowledge and access to PC. The authors are making efforts to initiate a PC nursing education and research consortium with the main goal of promoting PC and EOLC education and research in Oman. These and other efforts are needed in Oman and other low- and middle-income countries to promote access to quality PC and EOLC.

## Conclusion

To our knowledge, this is the first study to describe PC knowledge and attitudes towards EOLC in nurses working in Oman. The nurses working in tertiary hospitals in Oman have moderate PC and positive attitudes towards EOLC, highlighting the importance of tailored educational interventions and practical experiences to enhance the competencies and attitudes needed to deliver quality PC to patients and their families. By addressing the knowledge gaps identified by our study and promoting positive attitudes towards EOLC, the healthcare system in Oman can enhance the quality of end-of-life care and ultimately improve patient outcomes.
